# High Prevalence of Body Dysmorphic Disorder Among Rhinoplasty Candidates: Insights From a Cross-Sectional Study in Saudi Arabia

**DOI:** 10.7759/cureus.76431

**Published:** 2024-12-26

**Authors:** Almoaidbellah Rammal, Jamila S Bukhari, Ghadi N Alsharif, Haneen S Aseeri, Ameera A Alkhamesi, Rehaf F Alharbi, Wasan F Almohammadi

**Affiliations:** 1 Otolaryngology: Head and Neck Surgery, King Abdulaziz University, Jeddah, SAU; 2 Faculty of Medicine, King Abdulaziz University, Jeddah, SAU

**Keywords:** aesthetic surgery, bdd, body dysmorphic disorder, intervention strategies, prevalence, psychological screening, rhinoplasty

## Abstract

Background

Body dysmorphic disorder (BDD) is a psychiatric disorder characterized by excessive preoccupation with a perceived defect in one's appearance. Patients with BDD often seek cosmetic surgery to correct their perceived defect. Rhinoplasty is one of the most requested cosmetic surgeries. Our study aims to determine the prevalence of BDD among patients desiring rhinoplasty.

Methods

In a cross-sectional study using the Body Dysmorphic Disorder Questionnaire-Aesthetic Surgery (BDDQ-AS), we evaluated BDD prevalence in 340 participants who expressed a desire for rhinoplasty.

Results

We found that among 340 participants, 143 (43.3%) expressed a desire for rhinoplasty, with the majority being females (83.8%), and a prevalence of BDD among participants was 37.6%.

It was also observed that the willingness to have rhinoplasty was significantly associated with BDD (p < 0.001), with 53.1% of participants expressing a wish for rhinoplasty screening positive for BDD.

Our findings underscore the need for BDD screening in rhinoplasty candidates to prevent dissatisfaction and improve postoperative outcomes.

Logistic regression analysis identified significant predictors of BDD, with female gender demonstrating a substantial impact (OR = 4.26, 95% CI: 1.99-10.3, p < 0.001). This means that females are over four times more likely to experience BDD compared to males, highlighting a critical gender disparity. Similarly, the desire for rhinoplasty was strongly associated with BDD prevalence (OR = 3.45, 95% CI: 2.15-5.59, p < 0.001), indicating that individuals seeking rhinoplasty are more than three times as likely to have BDD compared to those without this desire. These findings underscore the importance of gender-specific screening and psychological evaluation, particularly for patients considering rhinoplasty, to identify and address potential body image concerns effectively.

Conclusion

Our study highlights the critical need for integrating mental health assessments into the preoperative process and standard practice for all rhinoplasty candidates to identify potential psychological concerns early. However, follow-up mental health support should be reserved for those who exhibit significant dissatisfaction with the results or show persistent BDD symptoms. It shows that BDD prevalence among individuals desiring rhinoplasty was high at 53.1%, and individuals who have a desire for rhinoplasty were 3.45 times more likely to screen positive for BDD than those with no desire.

## Introduction

Body dysmorphic disorder (BDD) is a condition where patients have concerns with their body image because of a defect in their appearance, which can be accurate but interpreted disproportionately or even non-existent but imagined by the patient. Those concerns may cause significant distress or functional damage to the patients that may extend to suicide [1,2].

BDD is not an uncommon disorder in our population. A study showed that the prevalence of BDD in the general Saudi population was 8.8%, and they found that this rate is considered higher than in the United States and Germany [3].

Patients may spend hours gazing at the mirror and concentrating on their images. Because the nose is the most prominent anatomical part of the face and has a central position, it can disturb the image of the body and personality development. So, it’s important at the psychological level [4].

Aesthetic operations have become very popular over the past two decades [5]. Rhinoplasty is one of the most requested aesthetic surgeries [6]. The prevalence of BDD among rhinoplasty candidates has been found to be high [7]. Many BDD patients may present to ear, nose, and throat; plastic; maxillofacial surgeons; and dermatologists to correct the perceived cosmetic defect, hoping that it will improve their quality of life [8]. This disorder can affect the outcome of the surgery as well, as the dissatisfaction rate will be high to the extent that some patients will repeat the surgery many times. Various studies showed that BDD patients who underwent cosmetic rhinoplasty have a low degree of satisfaction and postoperative worsening of BDD symptoms [9].

We emphasize the need to screen for body dysmorphic disorder (BDD) in rhinoplasty patients, as the demand for rhinoplasty is increasing in Saudi Arabia but has not been sufficiently studied in relation to psychiatric factors. Research into the potential link between rhinoplasty requests and body dysmorphic disorder is needed to better understand this trend. As the Australian Health Practitioner Regulation Agency (AHPRA) has recently made it mandatory for cosmetic surgery providers to conduct this screening [10], this policy shift highlights the growing recognition of mental health's critical role in surgical outcomes. Mandatory BDD screening could reduce post-surgical dissatisfaction rates and help avoid unnecessary surgeries, improving patient safety and satisfaction. In this study, we aim to evaluate the prevalence of BDD in patients desiring rhinoplasty.

## Materials and methods

Study design

This cross-sectional study was conducted using the Body Dysmorphic Disorder Questionnaire-Aesthetic Surgery (BDDQ-AS) [11]. Initially, we received 389 responses from otolaryngology clinic patients, but the total responses were 340 after exclusion. The study spanned from October 30, 2023, to January 17, 2024.

Inclusion and exclusion criteria

The selection criteria included patients of both sexes seeking rhinoplasty at otolaryngology clinics.

Patients aged 18 years or older and seeking rhinoplasty were included. Patients undergoing cosmetic surgeries other than rhinoplasty and undergoing functional rhinoplasty were excluded from the study.

Data collection

Data was gathered through the BDDQ-AS, a validated seven-item questionnaire. The survey evaluated participants for BDD based on responses indicating appearance concerns, preoccupations, and functional impairments. Data were collected using online platforms.

The survey consisted of seven questions. Positive screening for BDD is indicated when a patient affirms on the BDDQ-AS that they express apprehension regarding their appearance (affirmative response to question 1), exhibit preoccupation with these concerns (affirmative response to question 2), and experience at least moderate distress or impairment in various aspects of daily life due to these concerns (any affirmative response to questions 3, 4, 5, or 6 with a score of ≥ 3, or an affirmative response to question 7).

Statistical analysis

Statistical analysis was conducted using RStudio (R version 4.3.1, RStudio: Integrated Development Environment for R. Boston, MA. Retrieved from http://www.rstudio.com/). We expressed categorical variables as frequencies and percentages. Factors associated with BDD were investigated using Pearson's chi-squared or Fisher's exact test whenever applicable. The significantly associated variables were further incorporated into a binary logistic regression model to assess the predictors of BDD. Results were expressed as odds ratios (ORs) and 95% confidence intervals (95% CIs). Statistical significance was deemed at p < 0.05.

Ethical approval 

The study was conducted and approved by the Institutional Research Board at King Abdulaziz University, Jeddah, Saudi Arabia (approval number: HA-02-J-008). Participants were informed of the study's objectives and voluntarily agreed to participate, providing informed consent prior to completing the questionnaire.

## Results

Sociodemographic characteristics

Initially, we received 389 responses on the online platform. However, we excluded six reactions from those who disagreed to participate and 43 responses from those who had undergone rhinoplasty before. Therefore, we analyzed the data of 340 participants in the current study. The majority of participants in the study were between the ages of 18 and 23 years, 135 (39.7%), with a considerable representation from the 24 to 29 age group, 110 (32.4%). Females comprised the more significant proportion of the sample, accounting for 83.8% of the participants. Regarding educational attainment, a considerable portion held a bachelor's degree, 247 (72.6%). Regarding marital status, the majority identified as single: 262 (77.1%). Notably, 43.3% of the participants wanted to undergo rhinoplasty (Table 1).

**Table 1:** Sociodemographic characteristics

Characteristic	Missing	N (%)
Age	0 (0%)	
< 18 years		42 (12.4%)
18 to 23 years		135 (39.7%)
24 to 29 years		110 (32.4%)
> 29 years		53 (15.6%)
Gender	0 (0%)	
Male		55 (16.2%)
Female		285 (83.8%)
Educational level	0 (0%)	
Elementary school		1 (0.3%)
Middle school		2 (0.6%)
High school		90 (26.5%)
Bachelor's degree		247 (72.6%)
Marital status	0 (0%)	
Single		262 (77.1%)
Married		65 (19.1%)
Divorced		13 (3.8%)
Wish to do rhinoplasty	10 (2.9%)	
No		187 (56.7%)
Yes		143 (43.3%)

Participants’ responses to the BDDQ-AS survey

In general, reliability analysis showed excellent internal consistency (Cronbach’s alpha = 0.892, seven items). The participants' responses revealed that a significant portion reported being very worried about their appearance, 205 (60.3%). Additionally, 147 (43.2%) indicated that these concerns preoccupy their thoughts, with 95 (27.9%) experiencing distress at a mild level, 84 (24.7%) at a moderate level, and 22 (6.5%) at a severe level. Concerns also caused impairment in social, occupational, or other essential functioning for 145 (42.6%) of participants, with varying degrees of severity. Around 152 (44.7%) of participants noted interference with social life, with 100 (29.5%) reporting interference with schoolwork, job, or role functioning. Notably, 103 (30.3%) of participants reported avoiding activities or places due to these concerns (Table 2).

**Table 2:** Participants’ responses to the BDDQ-AS survey BDDQ-AS: Body Dysmorphic Disorder Questionnaire-Aesthetic Surgery

Characteristic	N (%)
Are you very worried about your appearance in any way (like the appearance of your nose, hair, or skin)	
No	135 (39.7%)
Yes	205 (60.3%)
Do these concerns preoccupy you? That is, do you think about it a lot and do you wish you could worry about it less?	
No	193 (56.8%)
Yes	147 (43.2%)
Did these concerns cause you a lot of distress torment or pain?	
No	121 (35.6%)
Mild, not too disturbing	95 (27.9%)
Moderate, disturbing but still manageable	84 (24.7%)
Severe, very disturbing	22 (6.5%)
Extreme disabling	18 (5.3%)
Did these concerns cause you impairment in social, occupational, or other important functioning?	
No	195 (57.4%)
Mild, not too disturbing	62 (18.2%)
Moderate, disturbing but still manageable	54 (15.9%)
Severe, very disturbing	16 (4.7%)
Extreme disabling	13 (3.8%)
Did these concerns often significantly interfere with your social life?	
No	188 (55.3%)
Mild, not too disturbing	64 (18.8%)
Moderate, disturbing but still manageable	56 (16.5%)
Severe, very disturbing	21 (6.2%)
Extreme disabling	11 (3.2%)
Did these concerns often significantly interfere with your schoolwork, job, or ability to function in your role?	
No	240 (70.6%)
Mild, not too disturbing	37 (10.9%)
Moderate, disturbing but still manageable	41 (12.1%)
Severe, very disturbing	17 (5.0%)
Extreme disabling	5 (1.5%)
Are there things you avoid because of these concerns (like going to any place, or being with anyone)?	
No	237 (69.7%)
Yes	103 (30.3%)

BDD prevalence and the associated factors

**Figure 1:**
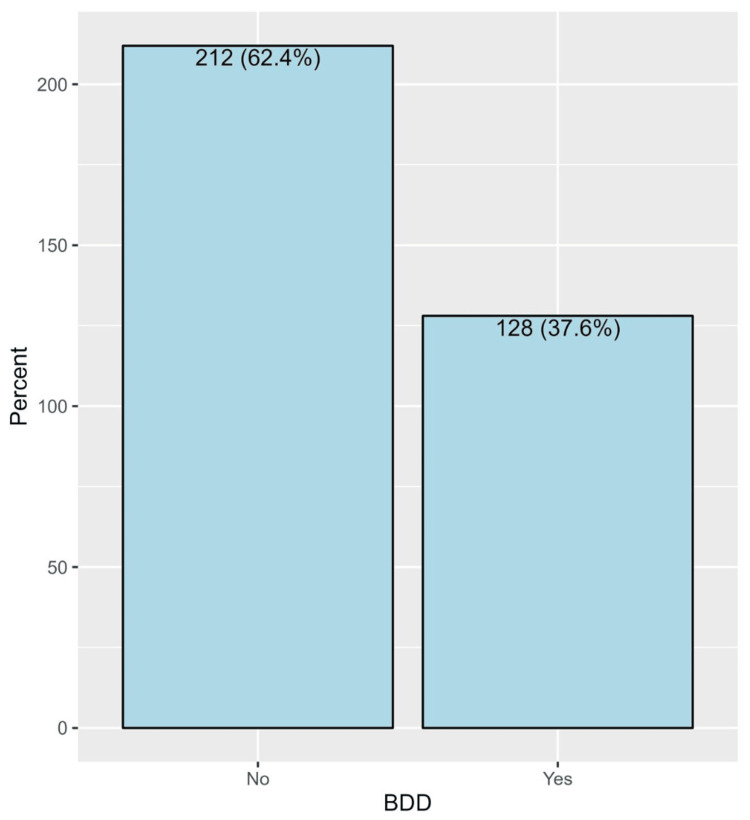
The prevalence of BDD among participants BDD: body dysmorphic disorder

The prevalence of BDD among participants was 37.6% (Figure 1). Gender exhibited a significant association (p < 0.001), revealing that a higher percentage of females (42.1%) screened positive for BDD in comparison to males (14.5%). Furthermore, the desire to undergo rhinoplasty was significantly associated with BDD (p < 0.001), with 53.1% of participants expressing a wish for rhinoplasty screening positive for BDD, as opposed to 25.1% among those not desiring rhinoplasty. Other sociodemographic characteristics were not significantly associated with BDD (Table 3).

**Table 3:** Factors associated with body dysmorphic disorder (BDD) among participants The data presented were analyzed using the chi-square test. The variables include age, gender, educational level, marital status, and the desire to undergo rhinoplasty.

Characteristic	Missing	BDD			p-value
		No N=212	Yes N=128	Test Statistics	
Age	0 (0%)			Chi-square=2.89	0.187
< 18		30 (71.4%)	12 (28.6%)		
18 to 23		76 (56.3%)	59 (43.7%)		
24 to 29		74 (67.3%)	36 (32.7%)		
> 29		32 (60.4%)	21 (39.6%)		
Gender	0 (0%)			Chi-square=13.45	<0.001
Male		47 (85.5%)	8 (14.5%)		
Female		165 (57.9%)	120 (42.1%)		
Educational level	0 (0%)			Chi-square=0.74	0.836
Elementary school		1 (100.0%)	0 (0.0%)		
Middle school		2 (100.0%)	0 (0.0%)		
High school		55 (61.1%)	35 (38.9%)		
Bachelor's degree		154 (62.3%)	93 (37.7%)		
Marital status	0 (0%)			Chi-square=2.11	0.521
Single		161 (61.5%)	101 (38.5%)		
Married		44 (67.7%)	21 (32.3%)		
Divorced		7 (53.8%)	6 (46.2%)		
Wish to do rhinoplasty	10 (2.9%)			Chi-square=24.67	<0.001
No		140 (74.9%)	47 (25.1%)		
Yes		67 (46.9%)	76 (53.1%)		

The logistic regression analysis aimed at identifying predictors of BDD revealed significant associations for two key characteristics. Female gender was found to have a substantial impact, with a notable odds ratio (OR) of 4.26 (95% CI: 1.99, 10.3, p < 0.001), indicating that females were 4.26 times more likely to screen positive for BDD compared to males. Additionally, the desire to undergo rhinoplasty emerged as a significant predictor, with an OR of 3.45 (95% CI: 2.15, 5.59, p < 0.001), signifying that individuals expressing a wish for rhinoplasty were 3.45 times more likely to screen positive for BDD than those who did not express such a desire (Table 4).

**Table 4:** Logistic regression analysis of predictors for body dysmorphic disorder (BDD) among rhinoplasty candidates The statistical test used for analysis is logistic regression. The table presents the odds ratio (OR), 95% confidence interval (CI), and p-value for the predictors of BDD among participants. The predictors include gender and the desire for rhinoplasty.

Characteristic	Odds Ratio	95% Confidence Interval	Test Statistics	p-value
Gender				
Male	Reference	Reference		
Female	4.26	1.99, 10.3	Z=4.15	<0.001
Wish to do rhinoplasty				
No	Reference	Reference		
Yes	3.45	2.15, 5.59	Z =5.07	<0.001

## Discussion

To our knowledge, this study marks the first attempt to assess the prevalence of body dysmorphic disorder (BDD) among individuals desiring rhinoplasty using the BDDQ-AS.

A notable 143 (43.3%) of participants expressed a desire for rhinoplasty, with a majority being female, 285 (83.8%), aligning with findings from Iran [5,12]. Furthermore, 262 (77.1%) of the sample were single, and a considerable portion, 135 (39.7%), belonged to the 18-23 age group, with 247 (72.6%) holding a bachelor's degree.

A significant 205 (60.3%) of participants were concerned about their appearance, echoing previous studies where 39% of BDD-affected individuals were specifically concerned about their noses [13]. These concerns were more than fleeting, with 43.2% of participants reporting persistent preoccupation, in contrast to a lower rate of 21.5% in another study [14]. This highlights the potential for such worries to disrupt daily life. Additionally, 95 (27.9%) experienced mild distress due to these concerns, underlining the emotional impact.

These concerns about appearance were not confined to mere thoughts and feelings; they had tangible effects on various aspects of participants' lives. A considerable majority, accounting for 145 (42.6%) of participants, reported impairment in social or occupational functioning due to their worries.

This impairment manifested in different domains, with 152 (44.7%) mentioning interference with their social life. In contrast, a similar study reported that 65% of participants also avoid social situations [15], and 100 (29.5%) reported impacts on their job, school, and functioning. Moreover, a significant portion, 103 (30.3%), admitted to actively avoiding activities or places because of their appearance-related concerns, consistent with a previous finding [15] highlighting the extent to which these worries can dictate behavior and lifestyle choices.

The study also examined the prevalence of BDD among participants, which showed that 128 (37.6%) of them screened positive. Comparable studies utilizing the BDDQ-AS conducted in Belgium and the United States demonstrated elevated rates of BDD among patients seeking rhinoplasty, with prevalence rates of 47% and 32%, respectively [16,17].

Additionally, the observed prevalence of BDD in this study is notably higher than that reported among patients in dermatology clinics (9.5%) [18] and plastic surgery clinics for abdominoplasty (21.2%) [1].

Notably, there was a higher prevalence among females, with 42.1% screening positive, which is aligned with another study that similarly found a greater incidence of BDD among females according to the BDDQ [19], thereby underscoring potential gender differences in the manifestation of BDD.

Moreover, several studies exploring the risk factors associated with BDD consistently highlighted its higher prevalence among females [3].

Additionally, the study found a significant association between the desire for rhinoplasty and BDD. A striking 76 (53.1%) of participants expressing a wish for rhinoplasty screened positive for BDD, compared to only 47 (25.1%) among those who did not desire rhinoplasty. This association suggests that the desire for rhinoplasty may be intertwined with underlying psychological factors related to body image and self-esteem.

Furthermore, the study revealed significant gender disparities in the likelihood of screening positive for BDD. Females were 4.26 times more likely than males to experience body image-related distress, indicating a disproportionate burden of body image-related distress among women. 

Similarly, individuals expressing a desire for rhinoplasty were 3.45 times more likely to screen positive for BDD than those without such a desire, confirming the results of a study conducted in Iran [5] and highlighting the potential psychological complexities underlying rhinoplasty decisions.

Recommendations 

Based on our study's insights, we recommend further investigating how cultural and societal norms impact BDD, especially in areas with a high demand for cosmetic surgeries. This research could lead to tailored screening and intervention strategies. Additionally, assessing the impact of mental health support before and after cosmetic procedures; for example, those with BDD or at risk may undergo a series of cognitive behavioral therapy (CBT) sessions to explore and address their belief that their nose is "flawed" or "ugly." Post-surgery, the patient may continue CBT to assess satisfaction with the outcome and reduce the risk of developing further obsession with their appearance; such measures are crucial for enhancing the treatment outcome in patients.

Limitations and strengths

While this study offers valuable insights into the relationship between BDD and rhinoplasty, it is essential to acknowledge its limitations. First, it used a self-structured questionnaire distributed through social media platforms, so people without access to those applications may find it challenging to participate in this research.

Additionally, there's a risk of sampling bias influencing this study's findings, as the sample population is not representative, potentially leading to overestimation or underestimation of BDD prevalence, particularly the predominance of female participants (83.8%) in our study. Thus, future studies should prioritize including a more balanced gender representation to ensure a comprehensive understanding of the psychological and demographic factors influencing rhinoplasty demand.

Moreover, reliance on self-reported data for assessing BDD prevalence could introduce inaccuracies due to social desirability bias or misunderstanding of diagnostic criteria. Despite these limitations, the study's strengths are notable. It addresses a gap in the literature by focusing on BDD prevalence specifically in this population, offering valuable clinical insights for otorhinolaryngologists and plastic surgeons, and potentially improving patient outcomes by facilitating appropriate management strategies and reducing postoperative dissatisfaction. Furthermore, the study lays a foundation for further research exploring the relationship between BDD and cosmetic surgery outcomes, as well as the effectiveness of interventions aimed at addressing BDD symptoms in this context.

## Conclusions

This study highlights the importance of addressing the psychological dimensions of cosmetic surgery, particularly rhinoplasty, where body dysmorphic disorder (BDD) is highly prevalent. Screening for BDD in rhinoplasty candidates is essential to identify those at risk of postoperative dissatisfaction and provide appropriate mental health support. Integrating psychological evaluations into preoperative assessments can improve patient care and outcomes. The strong association between the desire for rhinoplasty and BDD underscores the need for tailored, patient-centered approaches. Gender differences in BDD prevalence call for a gender-sensitive clinical focus. Collaboration between surgeons and mental health professionals is crucial to ensuring holistic care. Raising awareness about BDD can reduce stigma, promote early intervention, and enhance ethical practices in aesthetic surgery. This study provides a foundation for improving surgical outcomes and guiding future research into BDD and cosmetic procedures.
